# Endothelial Cell Responses to a Highly Deformable Titanium Alloy Designed for Vascular Stent Applications

**DOI:** 10.3390/jfb12020033

**Published:** 2021-05-14

**Authors:** Raluca Ion, Gaëtan Cabon, Doina-Margareta Gordin, Elena Ionica, Thierry Gloriant, Anisoara Cimpean

**Affiliations:** 1 Department of Biochemistry and Molecular Biology, Faculty of Biology, University of Bucharest, 91-95 Splaiul Independentei, 050095 Bucharest, Romania; rciubar@yahoo.com (R.I.); elena.ionica@g.unibuc.ro (E.I.); 2 University of Rennes, INSA Rennes, CNRS, Institut des Sciences Chimiques de Rennes—UMR 6226, F-35000 Rennes, France; gaetan.cabon@insa-rennes.fr (G.C.); doina.gordin@insa-rennes.fr (D.-M.G.); thierry.gloriant@insa-rennes.fr (T.G.)

**Keywords:** titanium alloy, in-vitro biocompatibility, endothelial cells, vascular stents

## Abstract

Titanium alloys are widely used for biomedical applications due to their good biocompatibility. Nevertheless, they cannot be used for balloon expandable stents due to a lack of ductility compared to cobalt-chromium (Co-Cr) alloys and stainless steels. In this study, a new highly deformable Ti-16Nb-8Mo alloy was designed for such an application. However, the biological performance of a stent material is strongly influenced by the effect exerted on the behavior of endothelial cells. Therefore, the cellular responses of human umbilical vein endothelial cells (HUVECs), including morphological characteristics, cell viability and proliferation, and functional markers expression, were investigated to evaluate the biocompatibility of the alloy in the present study. The in vitro results demonstrated the suitability of this alloy for use as endovascular stents.

## 1. Introduction

Currently, balloon expandable vascular stents are usually made of cobalt-chromium (Co-Cr) alloys or stainless steels which combine necessary requirements for such medical devices: a high elongation associated with a high tensile strength [1,2]. Even though these alloys exhibit suitable mechanical properties, biocompatibility is a major concern for such alloys containing a high proportion of Cr and Ni elements. Previous studies evaluated the biocompatibility response of several elements and metals. These studies pointed out that the corrosion resistance of cobalt-chromium alloys and stainless steels is directly related to their Ni and Cr concentrations. They may induce hydrolysis, redox, and complex metal ion-organic molecule binding reactions. For titanium (Ti) elements, none of these reactions was seen [3,4]. Ti-based alloys are well known to resist corrosion and to be biocompatible. These properties allow them to be used for biomedical applications such as for dental implants and orthopedic devices. One of the main Ti-based alloys used for such applications is the Ti-6Al-4V ELI alloy [5,6,7,8,9,10]. Even if these Ti-based medical alloys display an excellent long term hemocompatibility [11], they are not suitable for balloon expandable vascular stents because of their low plastic deformation capacity with a maximum tensile elongation around 15%. This value is thus much lower than those obtained with Co-Cr alloys or stainless steels (>40%) [1,2]. This lack of plastic deformation capacity of Ti alloys is well-known to be their major disadvantage for these biomedical applications. Indeed, they may lead to a stent fracture during their positioning in the artery or other dynamic failures. Consequently, designing new biocompatible Ti-based alloys with a high deformability became a new challenge. Recent studies have pointed out that the stress- or strain-induced martensitic (SIM) transformation occurring in metastable β titanium alloys can lead to combination of both a transformation-induced plasticity (TRIP) effect and a twinning-induced plasticity (TWIP) effect [12]. With this new type of Ti alloys, a high mechanical deformability can be reached. Therefore, we recently designed a new Ti-16Nb-8Mo alloy composition displaying remarkably high plastic deformation and strain-hardening, which is promising for the manufacture of balloon expandable vascular stents [13]. In this alloy, niobium (Nb) and molybdenum (Mo) were chosen as alloying elements because of their biocompatibilities. Indeed, Ti-Nb-Mo alloys clearly demonstrated exceptional corrosion resistance in simulated body fluids due to the spontaneous formation on their surface of a stable passivating oxide layer [14]. On the other hand, some studies have shown that Ti alloys containing Nb and Mo have mechanical compatibility and excellent cytocompatibility [15,16,17,18,19,20,21,22]. Nevertheless, the biological performance of a stent material is greatly influenced by the effect exerted on endothelial cell behavior. Indeed, for a rapid and complete endothelialization, stent material should support the growth, migration, and function of endothelial cells. Therefore, in the present study, we investigated the effects of Ti-16Nb-8Mo substrate on the human umbilical vein endothelial cells (HUVECs) responses, including cellular morphology, cytoskeleton organization, cell proliferation, and functional markers expression. For comparison, biomedical Co-Cr alloy and 316L stainless steel grades were used as material references.

## 2. Materials and Methods

### 2.1. Design and Characterization of the Alloy

**Figure 1:**
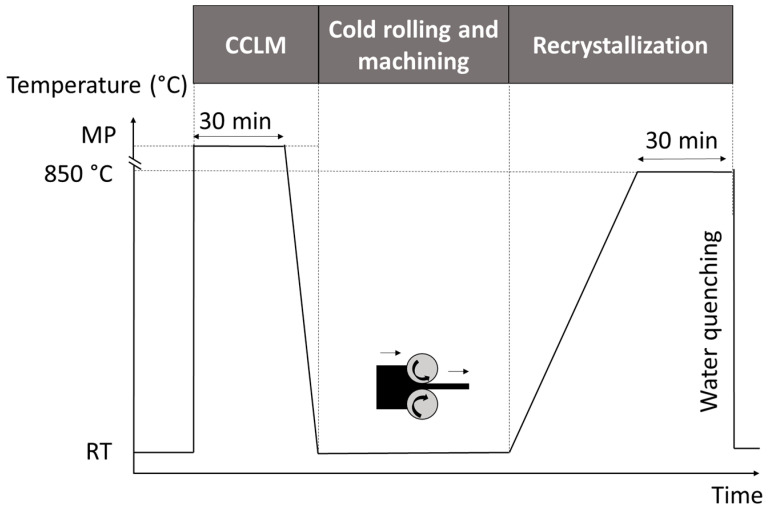
Thermo-mechanical treatments performed on the Ti-16Nb-8Mo alloy (MP stands for the melting point temperature used for the cold crucible levitation melting and RT corresponds to room temperature).

As shown in Figure 1, cold crucible levitation melting (CCLM) under pure Ar atmosphere was used to synthesize the Ti-16Nb-8Mo (wt.%) alloy. The CCLM technique is indeed very efficient to melt metals possessing high melting points to obtain ingots with a homogeneous composition without any problem of contaminations [23]. The ingots (of about 20 g) were then cold rolled to obtain 0.5 mm thick plates. Normalized dog-bone tensile specimens (3 mm wide, 0.5 mm thick, and 15 mm of gage length) and disc samples (10 mm diameter and 0.5 mm thick) were machined from the plates to carry out the mechanical and microstructural investigations and the in vitro biological assessments. All samples were heat treated under high vacuum at 850 °C for 30 min followed by a water quenching in order to retain the β-phase microstructure at room temperature. An energy-dispersive X-ray spectroscopy (EDS) analysis allowed us to verify the composition of the Ti-16Nb-8Mo alloy and a maximum 0.5 wt.% error was achieved corresponding to the EDS method accuracy.

To characterize the microstructure of the alloy, electron back-scattered diffraction analyses (EBSD detector, AZtecHKL system, Oxford Instruments, High Wycombe, UK) were carried out using a scanning electron microscope (SEM, JSM 7100F, Jeol, Tokyo, Japan). Prior to the EBSD observations, silicon carbide abrasive papers whose grid size was gradually finer were used and followed by a final polishing step with a colloidal silica suspension to obtain mirror-polished specimens. The colloidal silica suspension whose particle size was 0.05 µm was mixed with H_2_O_2_ solution to release stress due to polishing and reveal the microstructure. Tensile tests were performed to characterize the mechanical behavior of the alloys. The strain rate was 10^−4^ s^−1^ and the tensile direction was chosen parallel to the rolling one. To precisely measure the strain of a specimen, an extensometer was used.

### 2.2. In Vitro Endothelial Cell Response

#### 2.2.1. Cell Culture

Human umbilical vein endothelial cells (HUVECs) purchased from American Type Culture Collection (ATCC, Manassas, VA, USA) were incubated in Kaighn’s Modification of Ham’s F-12 Medium supplemented with 10% fetal bovine serum (Gibco, Grand Island, NY, USA), 1% penicillin—streptomycin (Gibco, Grand Island, NY, USA), and 30 μg/mL endothelial cell growth supplement (Sigma-Aldrich Co., St. Louis, MO, USA) at 37 °C in 5% CO_2_ atmosphere. Experiments were conducted using HUVECs at passages 4–6. HUVECs were seeded on the surfaces of the metal samples, placed in 24-well plates, at a density of 10^4^ cells/cm^2^ and maintained for up to 72 h in standard culture conditions. Prior to cell seeding, samples were sterilized by soaking in 70% ethanol for 30 min. Then, the samples were rinsed twice for 30 min in sterile-filtered MilliQ water, air dried, and exposed to ultraviolet light in a sterile tissue culture hood for 30 min on each side.

#### 2.2.2. Endothelial Cell Morphology

Morphologies of HUVECs cultured on test samples were observed by fluorescence microscopy. Briefly, at 24 h and 72 h after seeding, the cells were fixed with 4% paraformaldehyde (in phosphate buffered saline—PBS) for 20 min, permeabilized by incubation with 0.1% Triton X-100/2% bovine serum albumin (BSA) for 15 min at room temperature, stained with Alexa Fluor 488 Phalloidin (20 µg/mL, Invitrogen, Eugene, OR, USA) for 15 min to label cytoskeletal filamentous actin (F-actin), and counterstained with 4′-6-diamidino-2-phenylindole (DAPI) (Sigma-Aldrich Co., St. Louis, MO, USA) for 15 min to label the cell nuclei. Thereafter, representative images observed with an inverted fluorescence microscope (Olympus IX71, Olympus, Tokyo, Japan) were captured using Cell F software [24] (Version 2.7, Olympus Soft Imaging Solutions, Münster, Germany).

#### 2.2.3. Cell Viability/Proliferation Assessment

To assess the potential of the test samples to support the cell viability and proliferation, LIVE/DEAD and CCK-8 (Cell Counting Kit-8, Sigma-Aldrich Co., St. Louis, MO, USA) assays were performed according to the manufacturers’ instructions. Thus, to distinguish live from dead cells, the cell-populated samples were stained with LIVE/DEAD Viability/Cytotoxicity Kit (L-3224, Molecular Probes, Eugene, OR, USA), as we previously reported [25]. The quantitative analysis of HUVECs proliferation was performed using the Cell Counting Kit-8 in accordance with the protocol already presented [26]. Briefly, after specified culture periods, HUVECs were incubated with 10% CCK-8 reagent for 2 h at 37 °C in a 5% CO_2_ atmosphere. The optical density (OD) was then measured at 450 nm using a microplate reader (FlexStation 3 Multi-Mode Microplate Reader, Molecular Devices, San Jose, CA, USA).

#### 2.2.4. Analysis of Expression of Endothelial Cell Markers

For the analysis of endothelial cell functional markers, e.g., VE-cadherin and von Willebrand factor, a previously reported investigation was performed [27] after 3 days of culture. Briefly, HUVECs in contact with test samples were fixed with 4% paraformaldehyde and permeabilized with 0.1% Triton X-100 in PBS. Nonspecific binding sites were blocked with 2% BSA. The cells were then incubated with primary antibody, anti-VE-cadherin, or anti-von Willebrand factor antibody (Santa Cruz Biotechnology, dilution 1:50 in 1.2% BSA in PBS) for 2 h at room temperature and then with secondary fluorophore conjugated antibodies (Alexa Fluor 488- or Alexa Fluor 546-conjugated goat anti-mouse antibody, Santa Cruz Biotechnology, dilution 1:200 in 1.2% BSA in PBS). The labeled cells were washed with PBS and counterstained with DAPI. Afterwards, representative images were taken on an inverted fluorescence microscope (Olympus IX71, Olympus, Tokyo, Japan) and captured using Cell F software (Version 5.0, Olympus Soft Imaging Solutions, Münster, Germany). The corrected total cell fluorescence (CTFC) was measured from 5 fields chosen randomly per sample to quantify the expression of VE-cadherin and von Willebrand factor. For this purpose, ImageJ software [28] (NIH, Bethesda, MD, USA) was used. Therefore, an outline was drawn around each cell and area, and mean fluorescence and integrated density were measured, along with several adjacent background readings. The corrected total cellular fluorescence was calculated by applying the following formula: CTFC = integrated density—(area of selected cell × mean fluorescence of background readings).

#### 2.2.5. Nitric Oxide Release Test

For nitric oxide (NO) detection, HUVECs were seeded onto samples at a density of 5 × 10^4^ cells/cm^2^. After 3 days of culture, the medium was collected and NO release was measured using Griess reagent (Promega, Madison, WI, USA), as previously described [25].

#### 2.2.6. Statistical Analysis

For the quantitative biological tests performed, the data collected from triplicate samples were expressed as means ± SEM (standard error of the mean). Statistical analysis was performed with the GraphPad Prism software [29] (Version 3.03, San Diego, CA, USA). One-way ANOVA followed by Bonferroni’s multiple comparison test was used to assess the statistical significance of results between groups. *p* values of less than 0.05 were considered statistically significant.

## 3. Results and Discussion

### 3.1. Alloy Microstructure and Tensile Test

**Figure 2:**
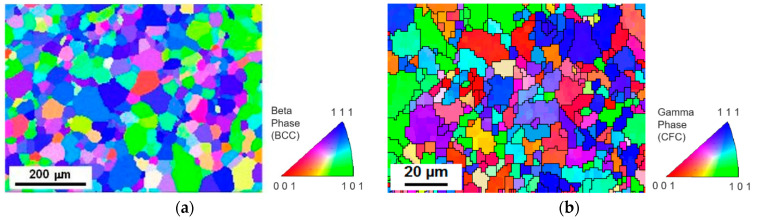
EBSD inverse pole figure (IPF) maps showing (**a**) the microstructure of the β-type Ti-16Nb-8Mo alloy, and (**b**) the γ austenitic microstructure of the Co-Cr alloy. Color code of the crystallographic orientations is indicated on the side.

After the elaboration and the thermo-mechanical treatment, the Ti-16Nb-8Mo alloy exhibited a single-phase equiaxed β-grain microstructure (body-centered cubic phase, BCC) as is shown on the EBSD inverse pole figure (IPF) map presented in Figure 2a. As is observed, the alloy was fully recrystallized, and the maximum grain size was about 100 µm. As a reference to the newly investigated Ti-16Nb-8Mo alloy, a Co-Cr alloy was studied concomitantly. In Figure 2b, the single-phase γ-austenitic microstructure (face-centered cubic phase, FCC) of the Co-Cr alloy with a 20 µm maximum grain size is shown. The color code corresponding to the crystallographic orientations of the different grains is indicated beside each EBSD map.

**Figure 3:**
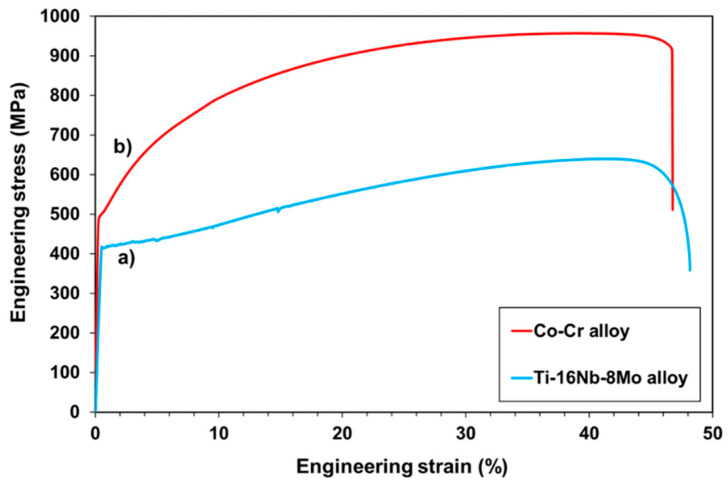
Engineering stress-strain tensile curves of (**a**) the Ti-16Nb-8Mo alloy (in blue), and (**b**) the Co-Cr alloy (in red).

Figure 3a presents a typical engineering stress-strain curve obtained for the recrystallized Ti-16Nb-8Mo alloy. It exhibited a 420 MPa yield stress and a 650 MPa ultimate tensile strength. As seen on the curve, an elongation higher than 45% was obtained (the strain at breaking is around 0.48). The Ti-16Nb-8Mo alloy can also be easily cold-rolled without being affected by noticeable damages. This is related to a good cold deformability. The engineering stress-strain curve of the Co-Cr alloy taken as reference in this study is presented in Figure 3b. It can be noted that the tensile curve of the Ti-16Nb-8Mo alloy clearly showed an elongation similar to that of the Co-Cr alloy although its tensile strength was lower. It is worth noting that the currently used Ti alloys in the biomedical field do not display such large plastic deformation. The exceptional elongation observed with the Ti-16Nb-8Mo alloy is due to a particular behavior inducing a TRIP-TWIP effect, which was recently highlighted [13]. Consequently, the newly designed Ti-16Nb-8Mo alloy containing only biocompatible elements, which displayed a high ductility with a 45% elongation, can become an attractive alloy for the manufacture of balloon expandable stents. It can overcome the major drawbacks of the currently used medical alloys: the poor plastic deformation of the current Ti-based medical alloys and the poor biocompatibility of the stainless steels and the Co-Cr alloys [1,2,6,8,11].

### 3.2. In Vitro Endothelial Cell Response

**Figure 4:**
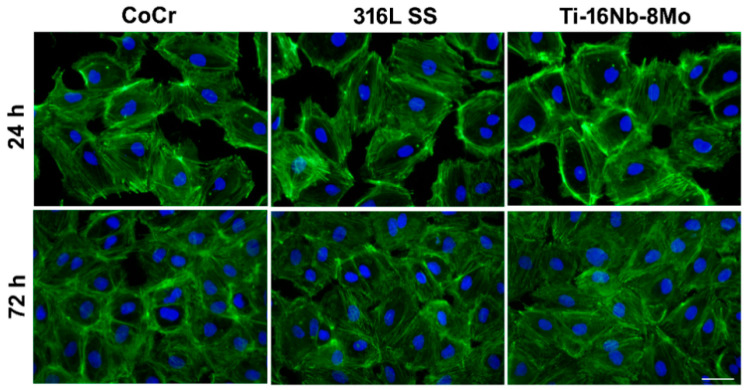
Fluorescence micrographs of HUVECs in contact with the Co-Cr, 316L SS, and Ti8Mo16Nb substrates for 24 h and 72 h. The cells are labeled for F-actin (green) and nucleus (blue). Scale bar represents 50 µm.

The quick endothelialization is a prerequisite for intravascular stents and could reduce the risk of restenosis and late thrombosis [30,31]. In line with this, as a potential stent material, Ti-16Nb-8Mo alloy should maintain the morphology, viability, proliferation, and functions of HUVECs. Consequently, in a first step of the study performed to assess the biocompatibility performance of the developed alloy, the morphological features of HUVECs were investigated through immunofluorescence staining of actin filaments by comparison with Co-Cr and 316L SS reference materials after 24 h and 72 h of culture (Figure 4). Our results revealed that HUVECs were able to adhere to and spread on all surfaces assuming the typical cobblestone-like morphology, with no significant morphological differences over time between the analyzed samples. Thus, HUVECs presented a normal cytoskeleton organization (ventral stress fibers and stress fibers arranged along the edges of each cell composed of actin filaments). Furthermore, the number of HUVECs on all analyzed substrates was significantly higher after 3 days of culture, covering a vast area of their surfaces and, thus, suggesting that all three materials can equally support the cell proliferation.

**Figure 5:**
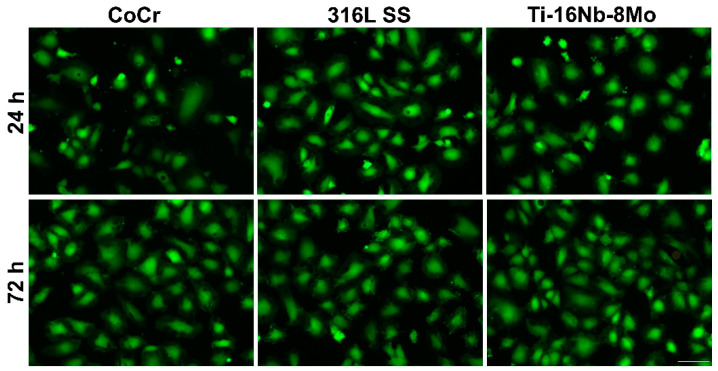
Fluorescence microscopy images of HUVECs grown in contact with the analyzed metallic substrates for 24 h and 72 h. Staining with LIVE/DEAD Cell Viability/Cytotoxicity Kit (live cells fluorescence green; no red fluorescent dead cells are present); Scale bar: 100 μm.

Next, the LIVE/DEAD assay revealed viable cells that converted the non-fluorescent calcein AM (acetoxymethyl ester) to green fluorescent calcein on all analyzed surfaces, both after 24 h and 72 h of culture (Figure 5). They displayed a typical morphology for endothelial cells and almost similar densities at a certain time point. Moreover, no red fluorescent dead cells and an increasing number of viable cells could be noticed over the culture period, suggesting a nearly equal capacity of the three metallic substrates to sustain cellular survival and proliferation.

**Figure 6:**
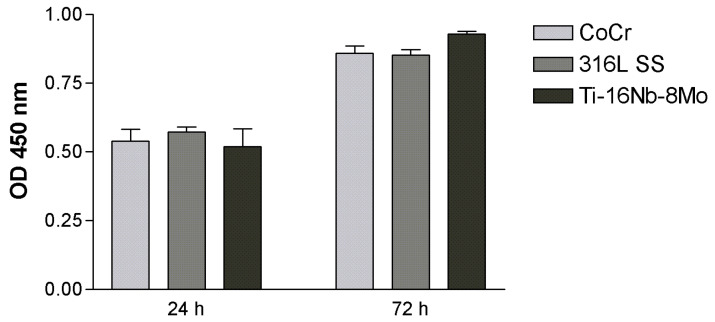
Proliferation of HUVECs as assessed by CCK-8 assay. Data analysis was based on mean ± SEM (n = 3); *p* > 0.05.

To finally assess the capacity of the developed Ti-16Nb-8Mo to support HUVECs viability and proliferation, the results of the LIVE/DEAD assay were combined with the quantitative data provided by the CCK-8. As can be seen in Figure 6, HUVECs showed a progressive growth over time in the case of all analyzed surfaces and this finding is consistent with the microscopic observation of the actin- and nuclei- labeled cells (Figure 4), as well as of the cells stained with the LIVE/DEAD kit (Figure 5). Moreover, at 3 days post-seeding, an increased proliferation rate on the Ti-based alloy was found as compared to the Co-Cr and 316L SS surfaces, though not statistically significant, suggesting its capacity to sustain post-implantation stent endothelialization.

**Figure 7:**
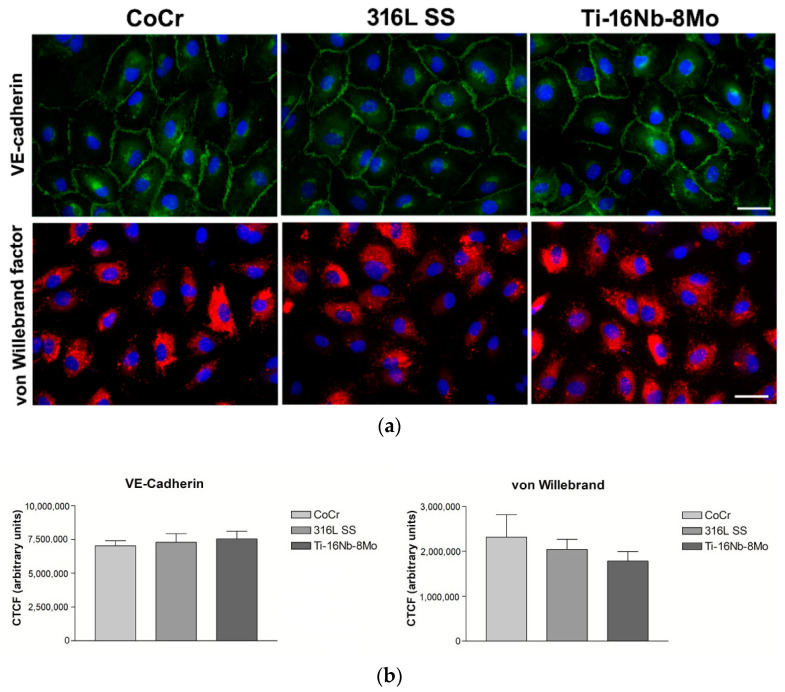
(**a**) Immunofluorescence images of HUVECs in contact with the analyzed samples. Staining with anti-VE-cadherin (green) and anti-von Willebrand factor (red). In each image, nuclei were labeled with DAPI (blue). Scale bar represents 50 µm. (**b**) Corrected total cellular florescence (CTFC) quantified by using ImageJ software. Results are represented as mean ± SEM (n = 3); *p* > 0.05.

In addition to endothelial cell morphology and proliferation, the effect of the new alloy on the expression of specific endothelial cell markers was evaluated. The maintenance of the endothelial cell phenotype was verified by the analysis of VE-cadherin and von Willebrand factor expression using immunofluorescence staining (Figure 7).

VE-cadherin is a strictly endothelial specific adhesion molecule located at junctions between endothelial cells, with a key role in the control of vascular permeability and leukocyte extravasation [32]. Moreover, VE-cadherin regulates various cellular processes, such as cell proliferation and apoptosis, and modulates vascular endothelial growth factor receptor functions [32,33]. As represented by the fluorescence micrographs (Figure 7a), VE-cadherin localized to cell–cell contacts and showed a continuous line of immunofluorescence describing the outer periphery of cells on all tested materials. Moreover, quantification by ImageJ of the specific fluorescence evidenced similar expression levels on the three analyzed metallic samples (Figure 7b). Likewise, HUVECs grown for 3 days onto test materials stained positive for von Willebrand factor showing a cytoplasmic punctate localization, mainly confined around the nucleus (Figure 7a). Notably, most cells grown in contact with Ti-16Nb-8Mo alloy exhibited bright red fluorescent signals specific to this marker for endothelial cells. It is acknowledged that von Willebrand factor is a multimeric protein that mediates adhesion of platelets to sites of vascular injury, often used to identify endothelial cells [34]. Considering that the corrected total cell fluorescence (CTFC) of the von Willebrand factor, measured from 5 microscopic fields per sample, displayed a descending trend in the order: Co-Cr > 316L SS > Ti-16Nb-8Mo, although without statistically significant differences between the samples, it can be concluded that the Ti-based alloy elicited the lower thrombogenic potential. Therefore, it shows promising potential for prospective stent applications.

**Figure 8:**
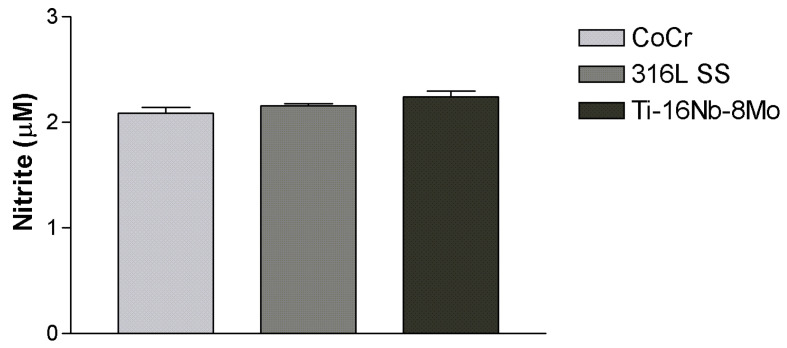
Nitrite concentrations in the culture media of HUVECs grown on test samples, as assessed by Griess reagent assay. Data are presented as mean ± SEM (n = 3); *p* > 0.05.

To further evaluate the function of HUVECs grown in contact with Ti-16Nb-8Mo surface, NO release was measured. NO is continuously secreted by endothelial cells and plays a critical role in maintaining vessel homeostasis by inhibiting platelet aggregation, preventing blood coagulation, and promoting angiogenesis [35]. The level of NO released by HUVECs grown on test substrates is shown in Figure 8. As can be seen, these cells were almost equally capable of secreting NO, suggesting the maintenance of their differentiated phenotype.

Altogether, these studies concerning the suitability of the Ti-16Nb-8Mo to be used for constructing endovascular stents demonstrated that this alloy exhibits in vitro cell behavior close to, even slightly superior to, Co-Cr and 316L SS materials, which are used in commercial stents. In contrast to these results, a previous study by Yeah et al. [36] showed that 316L SS and nitinol inhibit HUVECs normal growth and download the expression of the endothelial specific markers (endothelial nitric oxide synthase, von Willebrand factor, and connexin 43 protein). These contradictory data could be due to the differences in the cell seeding densities used in these two investigations.

These good results are in accordance with a previous in vitro study in which a noticeable biocompatibility of the Ti-16Nb-8Mo alloy with respect to the preosteoblast response was evidenced [37]. Consequently, this highly deformable Ti alloy can be envisaged for vascular stent applications from a biological point of view.

## 4. Conclusions

This study focused on a newly developed metastable β Ti-16Nb-8Mo alloy for medical applications. For balloon expandable stents, an alloy possessing a remarkably high mechanical deformability is required. Unlike the currently used Ti alloys in the biomedical field, the studied Ti-16Nb-8Mo alloy displayed such necessary property with a tensile elongation higher than 45%. On the other hand, the excellent biocompatibility of the alloy was evidenced by in vitro tests assessing the human umbilical vein endothelial cell (HUVEC) responses, including cellular morphology, cytoskeleton organization, cell viability/proliferation, and expression of the specific markers for endothelial cell phenotype. In conclusion, due to its high plastic deformation capacity and its excellent biocompatibility, the Ti-16Nb-8Mo alloy fulfils all the conditions to be used for the manufacture of balloon expandable stents compared with Co-Cr alloys and stainless steels.

## Data Availability

The data presented in this study are available on request from the corresponding author.
